# Cesium Accumulation Patterns and Stress Response in Hydroponic Radish (*Raphanus sativus* L.): A Physiological–Transcriptomic Study

**DOI:** 10.3390/plants14121802

**Published:** 2025-06-12

**Authors:** Yu-Han Wen, Xi Chen, Ming Sun, Chao-Hui Yang, Meng-Yuan Xu, Feng-Xiang Lai, Si-Qi Fu, Yu-Meng Fan, Xin-Peng Guo, Qun Li, Guo Wu

**Affiliations:** 1 College of Life Science, Sichuan Normal University, Chengdu 610101, China; morningstar-911@outlook.com (Y.-H.W.); ssun0311@163.com (M.S.); 15119915867@163.com (C.-H.Y.); 15282895943@163.com (M.-Y.X.); 18583005762@163.com (F.-X.L.); 18980717561@163.com (S.-Q.F.); 18328640034@163.com (Y.-M.F.); shixinpu19610613@163.com (X.-P.G.); liqun01234@163.com (Q.L.); 2 College of Resources and Environmental Sciences, Nanjing Agricultural University, Nanjing 210095, China; chenx_19981008@163.com

**Keywords:** cesium, photosynthesis, mineral elements, reactive oxygen species, antioxidant

## Abstract

The present study systematically investigated the cesium (Cs) enrichment characteristics and physiological responses to Cs exposure in radish (*Raphanus sativus* L.) seedlings under hydroponic conditions through integrated physiological, biochemical, and transcriptome analyses. The results showed that the Cs content in radish roots, stems, and cotyledons increased progressively with rising Cs concentrations (0.25–2 mM), and Cs mainly accumulated in the cotyledon. The transfer factor (TF) increased by 63.29% (TF = 3.87) as the Cs concentration increased from 0.25 to 2 mM, while the biological concentration factor (BCF) decreased by 72.56% (BCF = 14.87). Severe growth inhibition was observed at 2 mM Cs stress, with biomass reduction reaching 29.73%. The carotenoid content decreased by 11.92%; however, the total chlorophyll content did not change significantly, and the photosynthesis of radish was not affected. In addition, Cs exposure disrupted mineral nutrient homeostasis, decreasing potassium (K), sodium (Na), magnesium (Mg), and iron (Fe) content. The superoxide dismutase (SOD), peroxidase (POD), and catalase (CAT) activities, reactive oxygen species (ROS), and malondialdehyde (MDA) content increased under the different Cs treatments, which indicated that Cs exposure induced oxidative stress response in radish seedlings. Transcriptome analysis detected a total of 4326 differentially expressed genes (DEGs), in which altered expression patterns in genes associated with mineral transport, antioxidant systems, and carotenoid biosynthesis pathways in radish under 2 mM Cs treatment were observed. In conclusion, this study comprehensively investigated the physiological and molecular responses of radish to Cs stress, revealing that Cs accumulation exhibited site-specific preference and concentration dependence and induced physiological disturbances, including growth inhibition and photosynthetic pigment metabolism alterations. At the transcription level, Cs activated the enzymatic antioxidant system, related genes, and stress-response pathways. Notably, this study is the first to demonstrate that Cs disrupts plant mineral nutrition homeostasis and inhibits carotenoid biosynthesis. These findings establish a crucial theoretical foundation for utilizing radish in Cs-contaminated phytoremediation strategies.

## 1. Introduction

Cesium (Cs) is the known element with the strongest metallic properties, located in group I_A_ and in the sixth period; it has similar chemical properties to the congener K [1] and its background content is 8.24 mg·kg^−1^ in the cultivated soil layer of farmland in China [2]. At present, 39 isotopes of Cs have been found, among which ^133^Cs is the only stable isotope that naturally exists [3]; ^137^Cs (half-life_1/2_ = 30.2 y), as one of the common radionuclides, has become the focus of relevant researchers because it endangers public safety due to the discharge of Fukushima nuclear wastewater [4]. Traditional nuclide pollution treatment methods are mainly divided into physical methods (e.g., soil covering), chemical methods (e.g., extraction), and physicochemical methods (e.g., electrochemical treatment). These methods are usually expensive, inefficient, complicated, and have potential adverse effects on the environment [3]. As one of the popular methods to deal with radionuclides, heavy metals, and other pollutants in recent years, phytoremediation technology has been considered as a green, economic, and efficient method, which can be mainly divided into phytostabilization, rhizofiltration, phytodegradation, phytoextraction, and others [5,6,7,8]. Conventional mine tailings remediation techniques have been estimated to cost between USD 1.50 and USD 450 per m^3^, while phytoremediation techniques have been estimated to cost between USD 0.40 and USD 26 per m^3^ [9]. Therefore, phytoremediation technology is a great choice for Cs-contaminated soil.

Studies over the past decades have shown that the distribution pattern of Cs in plants has changed according to the plant species. Cs is enriched aboveground more than underground in *Brassica juncea* and *Cucumis sativus* [10,11,12]; the root is more enriched than the shoot in *Vicia faba* [12]. The aboveground part was larger than the underground part under the low Cs concentration treatment, but more Cs was found to accumulate in the underground part under the high Cs concentration treatment in *Amaranthus tricolor* [13]. It can be concluded that Cs enrichment in plants varies with plant species and even Cs concentration. A small amount of cesium ions (Cs^+^) can maintain electrolyte balance in plant cells, while excessive Cs^+^ can replace potassium ions (K^+^), leading to the inactivation of key components that require K^+^ in plants and seriously affecting plant growth and metabolism, e.g., a decrease in biomass, a reduction in root length and stem length, and it also damages the physiological functions of plants by inhibiting the absorption of K^+^ [11,14,15]. Cs affected the functions of ABCG37 and ABCG33 and other ABC transporter family proteins, and further influenced the absorption of Na^+^, Fe^2+^, and Mg^2+^ in plants, resulting in a decrease in Na^+^ content in *Arabidopsis thaliana* [16]. However, according to existing research, Cs stress has no significant effect on the Ca^2+^ content in *Plantago major* [17]. According to previous studies, there is no significant difference in the distribution characteristics of stable Cs and radioactive Cs in plants; therefore, ^133^Cs is commonly used to simulate ^137^Cs to explore the effects of Cs on plant growth and physiological responses [18,19,20]. The growth of *Nitella pseudoflabellata*, *Brassica juncea*, *Plantago major*, and *Arabidopsis rotundifolia* has been inhibited by Cs, inducing a decrease in plant biomass, cotyledon yellowing, and so on [15,17,21,22]. The chlorophyll content and the carotene were decreased significantly under Cs stress in *Cucumis sativus* [10] and *Nitella pseudoflabellata* [21]. Regarding the photosynthetic parameters’ response to cesium (Cs) stress, notable variations were observed among different plant species. Specifically, the maximum photochemical efficiency of photosystem II (Fv/Fm) in *Arabidopsis halleri* and *Plantago macrocarpa* exhibited a significant decline under Cs stress conditions [17,22]. In contrast, the Fv/Fm value of *Amaranthus tricolor* remained relatively stable [13]; however, the effective quantum yield of photosystem II (ΦPSII) and photochemical quenching coefficient (qP) decreased, while non-photochemical quenching (NPQ) increased significantly. Furthermore, Cs stress induced a substantial reduction in chlorophyll content, as well as in several key chlorophyll fluorescence parameters, including the ratio of variable to initial fluorescence (Fv/F0), Fv/Fm, ΦPSII, qP, and NPQ [23]. These findings collectively indicate that Cs exposure impairs the synthesis of photosynthetic pigments and disrupts the normal function of the photosynthetic apparatus, ultimately inhibiting plant growth and development. Such alterations in photosynthetic performance highlight the detrimental impact of Cs stress on plant physiological processes and underscore the importance of understanding plant responses to heavy metal contamination for ecological risk assessment and phytoremediation strategies. In terms of mineral metabolism, current studies mainly focus on the effect of Cs on K content, which generally leads to a decrease in it in plants [12,17,22,24,25]; however, there are few studies on the effect of Cs on the other mineral elements in plants. Both enzymatic and non-enzymatic antioxidant systems have been developed to maintain the homeostasis of reactive oxygen species (ROS) during long-term adaptation in plants. Cs stress usually causes oxidative stress in plants, which leads to a decrease in antioxidant enzyme activity, thereby destroying ROS homeostasis and causing peroxidation damage. Cs can induce the ROS burst in *Brassica juncea* [26]. A variety of plants, such as *Cucumis sativus* and *Atrichum undulatum*, produced oxidative stress in response to Cs treatment, and the activities of antioxidant enzymes increased [10,27].

The previous studies only focused on circumscribed investigation of Cs stress on a single physiological function, such as photosynthesis or antioxidant activities. Despite such reports, there have been few reports that comprehensively investigated the Cs enrichment characteristics and physiological responses to Cs exposure in plants under hydroponic conditions through integrated physiological, biochemical, and transcriptome analyses. Radish (*Raphanus sativus* L.), an annual herb of the cruciferous family, has been found to have the ability to hyperenrich metals such as Pb and Cd [28,29,30,31], and is an important economical vegetable crop [30]. Therefore, radish was selected as the experimental material in this paper, treated by 0–2 mM CsCl (^133^Cs), and then combined with physiological and biochemical experiments and transcriptome analysis to explore the enrichment characteristics and physiological responses of radish seedlings to Cs stress.

## 2. Results

### 2.1. The Radish Seedlings Growth Under Cs Stress

**Figure 1:**
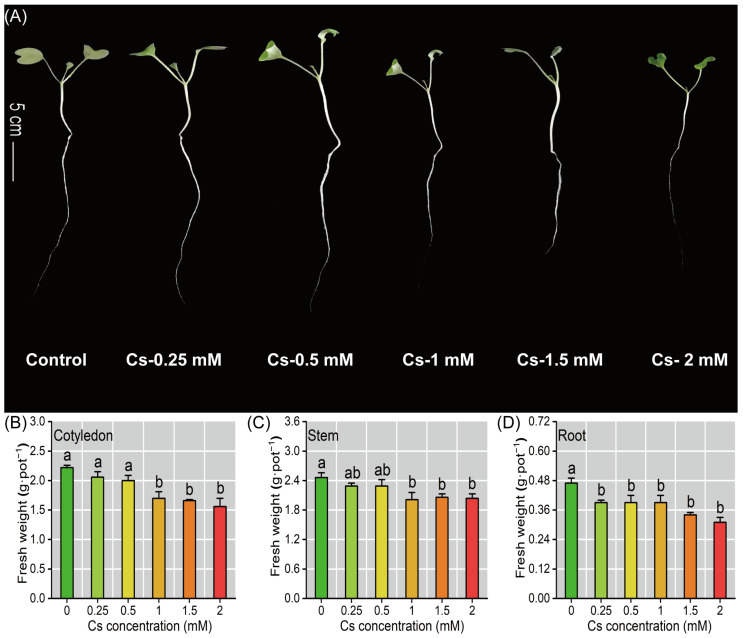
The growth of radish seedlings under different Cs exposure. (**A**) Phenotype of radish seedlings under Cs stress. Cs concentrations were 0, 0.25, 0.5, 1, 1.5, and 2 mM. (**B**–**D**) Cs reduced the biomass of radish seedlings’ roots, stems, and cotyledons. Different lowercase letters represent significant differences (*p* < 0.05) in treatment groups (*n* = 6).

Cs exposure exerted significant growth inhibitory effects on radish seedlings across all treatment concentrations, as evidenced by substantial reductions in both primary root elongation and cotyledon expansion compared to the controls (Figure 1A). The fresh weight of radish seedlings exhibited a significant reduction (*p* < 0.05) under the different Cs treatments, with the lowest values recorded at 2 mM Cs exposure; specifically, the mean fresh weights per plant decreased to 0.31 ± 0.02 g·pot^−1^ FW for roots (17.21–34.04%, *p* < 0.05), 2.04 ± 0.09 g·pot^−1^ FW for stems (6.91–17.07%, *p* < 0.05), and 1.56 ± 0.14 g·pot^−1^ FW for cotyledons (7.21–29.73%, *p* < 0.05) (Figure 1B–D).

### 2.2. Cs Accumulation Characteristics in Radish Seedlings

**Figure 2:**
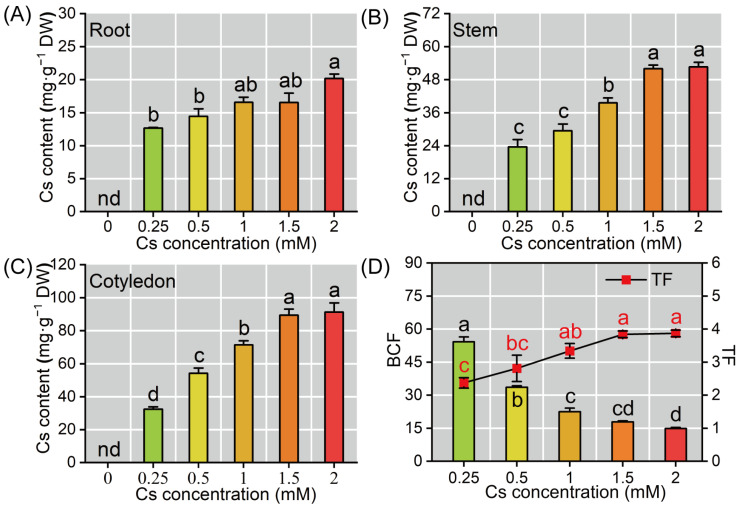
The enrichment characteristics of radish seedlings under different Cs treatments. (**A**–**C**) Cs accumulation in the root, stem, and cotyledon of radish seedlings. (**D**) The BCF and TF of Cs in radish seedlings. BCF indicates biological concentration factor, and TF indicates transfer factor. Different lowercase letters represent significant differences (*p* < 0.05) between the treatment groups (*n* = 6); nd means not detected.

The Cs accumulation in radish seedlings exhibited a concentration-dependent increase. Specifically, compared with the 0.25 mM Cs exposure, the Cs content increased by 13.97–59.11% in roots, 24.82–123.71% in stems, and 67.44–182.19% in cotyledons under 0.5–2 mM Cs treatments. Nonetheless, different plant organs exhibited distinct capacities for Cs enrichment; the highest Cs content was observed at 2 mM Cs exposure, with accumulation levels following the order: cotyledon (91.26 ± 5.6 mg·g^−1^ DW) > stem (52.64 ± 1.63 mg·g^−1^ DW) > root (20.16 ± 0.68 mg·g^−1^ DW) (Figure 2A–C), and exhibited a statistically significant difference (*p* < 0.05). These results suggest that aboveground tissues serve as the primary sites for Cs accumulation in radish. As Cs concentration increased, the biological concentration factor (BCF) of radish seedlings significantly decreased, while the transfer factor (TF) increased. Compared with the 0.25 mM Cs exposure, the TF increased by 63.29% (TF = 3.87) and the BCF decreased by 72.56% (BCF = 14.87) at the 2 mM Cs stress (Figure 2D); this might be related to the increased concentration of Cs causing a decrease in the radish biomass, which is expanded in the discussion.

### 2.3. Response of Photosynthetic Parameters of Radish Seedlings to Cs Stress in the Cotyledon

**Figure 3:**
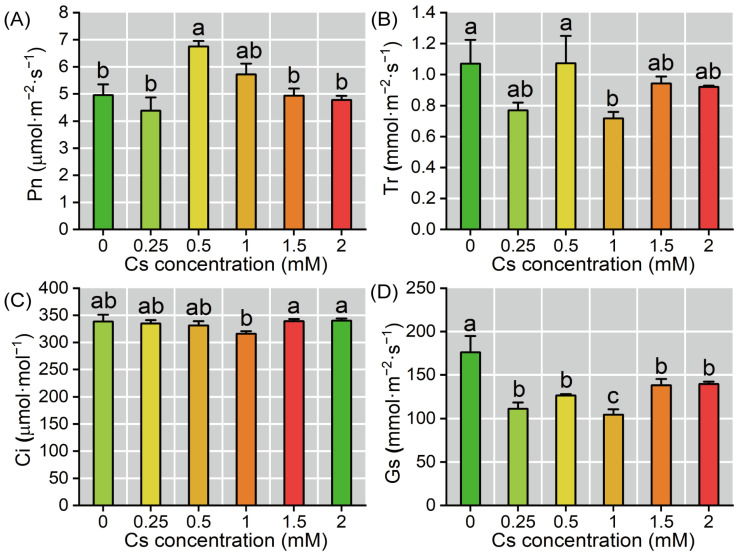
Cs affected photosynthetic gas exchange in the radish seedlings’ cotyledons. (**A**–**D**) Photosynthetic gas exchange parameters. Pn: net photosynthetic rate, Tr: transpiration rate, Ci: intercellular CO_2_ concentration, Gs: stomatal conductance. Different lowercase letters represent significant differences (*p* < 0.05) in treatment groups (*n* = 6).

Different concentrations of Cs exerted limited effects on the photosynthesis of radish cotyledon. Analysis of photosynthetic gas exchange parameters revealed that the Pn exhibited an increase (36.18%, *p* < 0.05) at 0.5 mM Cs concentration, and there were no significant differences between control and other treatments (Figure 3A). In comparison, Tr and Ci demonstrated lower sensitivity to Cs treatment, while Gs displayed a decreasing trend with increasing Cs concentration (20.62–40.73%, *p* < 0.05) (Figure 3B–D).

**Figure 4:**
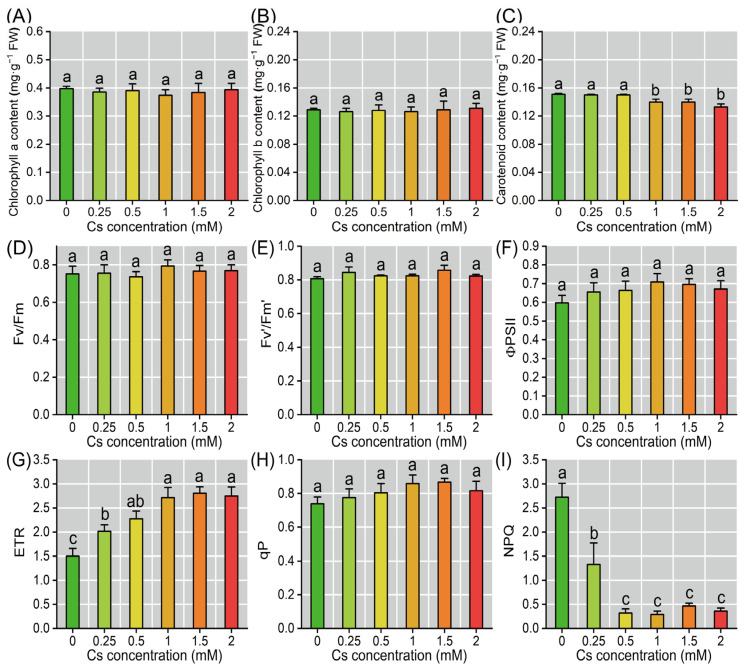
Cs affected chlorophyll fluorescence in the radish seedlings’ cotyledons. (**A**–**C**) Cs affected the pigment content of radish seedlings. Chl a: chlorophyll a, Chl b: chlorophyll b, Car.: carotenoid. (**D–I**) Chlorophyll fluorescence parameters. Fv/Fm: maximum photochemical efficiency, Fv’/Fm’: capture efficiency of excitation energy, ΦPSII: actual photochemical efficiency, ETR: electron transfer rate, qP: photochemical quenching coefficient, NPQ: non-photochemical quenching coefficient. Different lowercase letters represent significant differences (*p* < 0.05) in treatment groups (*n* = 6).

Photosynthetic pigment analysis revealed a concentration-dependent reduction in carotenoid content, which was observed at Cs concentrations ≥ 1 mM, exhibiting significant decreases ranging from 7.28% to 11.92% relative to the control plants (*p* < 0.05, Figure 4C). Carotenoids act as precursors for zeaxanthin synthesis. Their depletion may result in a decrease in zeaxanthin levels, thereby reducing NPQ and impairing downstream photoprotective effects. In contrast, there were no statistically significant alterations in chlorophyll a (0.394 ± 0.022 mg·g^−1^ FW) and chlorophyll b (0.131 ± 0.007 mg·g^−1^ FW) contents across experimental treatments (Figure 4A,B). Chlorophyll fluorescence parameters further indicated that key indices such as Fv/Fm, Fv’/Fm’, ΦPSII, and qP exhibited insensitivity to Cs treatment, with no significant changes observed. However, the ETR increased notably, reaching 1.34–1.87 times the control levels, while NPQ decreased significantly, ranging from 0.49 to 0.10 times the control values, as Cs concentration elevated (Figure 4D–I). These results collectively suggest that Cs treatment primarily influences stomatal conductance and may indirectly affect photosynthetic performance through changes in ETR and NPQ.

### 2.4. The Mineral Element Content Changes of Radish Seedlings Under Cs Stress

**Figure 5:**
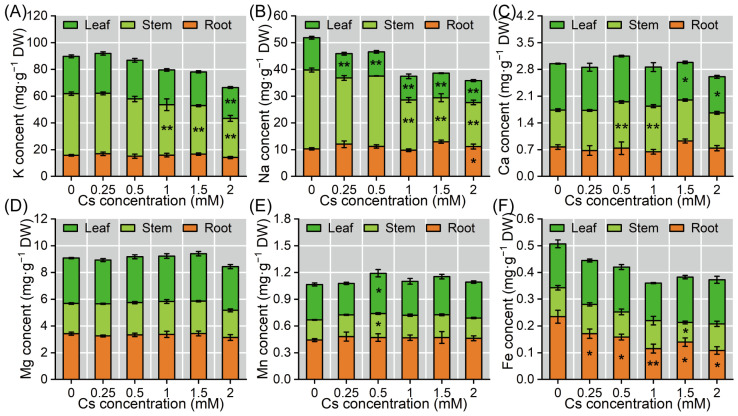
Cs disturbed mineral nutrient homeostasis in radish seedlings. (**A**–**F**) The K, Na, Ca, Mg, Mn, and Fe contents under different Cs exposures. * and ** represent significant differences in different treatment groups and the control at the 0.05 and 0.01 levels (*n* = 6).

Cs exposure significantly altered mineral homeostasis in radish seedlings, exhibiting organ-specific redistribution patterns (Figure 5). Compared with the control, potassium (K) content decreased by 2.10–36.71% in stems and 6.42–17.22% in cotyledons under different Cs concentrations (Figure 5A); sodium (Na) content increased by 7.84–25.70% in roots, while stems and cotyledons exhibited 10.59–44.09% and 24.31–32.47% decreases, respectively (Figure 5B). Calcium (Ca) homeostasis was differentially regulated, with stem concentrations increasing by 10.97–25.58% but cotyledon levels declining by 1.16–22.17% (Figure 5C). Magnesium (Mg) remained stable across all organs regardless of Cs treatment intensity (Figure 5D). When the concentration of Cs was 0.5 mM, manganese (Mn) displayed moderate increases of 0.23–18.91% in stems and 1.67–14.14% in cotyledons, whereas iron (Fe) experienced substantial depletion, particularly in roots (-27.12–54.02%) and stems (−3.23–32.18%) (Figure 5E,F). These results demonstrate that Cs stress preferentially disrupts the mineral metabolic homeostasis in shoot tissues, with K being the most severely affected macronutrient, followed by Fe in underground organs. The changes in the content of mineral elements may be related to the differential expression of the associated transport proteins. Ultimately, under the Cs exposure, the contents of K, Na, Mg, and Fe in the radish significantly decreased (*p* < 0.05), while the contents of Ca and Mn showed no significant change (*p* > 0.05).

### 2.5. Cs Induced Oxidative Stress in Radish Seedlings

**Figure 6:**
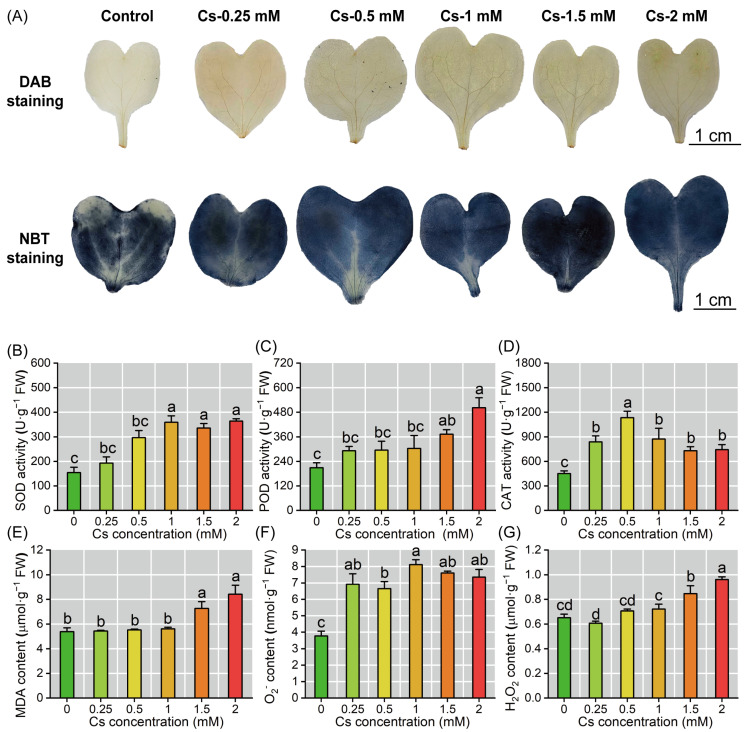
Cs disturbed ROS homeostasis in radish seedlings. (**A**) H_2_O_2_ and O_2_^−^ staining with DAB/NBT. (**B**) SOD activities. (**C**) POD activities. (**D**) CAT activities. (**E**) Malondialdehyde (MDA) contents. (**F**) O_2_^−^ content. (**G**) H_2_O_2_ content. The different lowercase letters represent significant differences (*p* < 0.05) in treatment groups (*n* = 6).

Cs exposure induced significant oxidative stress in radish seedlings. Histochemical staining and quantitative analysis revealed that exposure to varying Cs concentrations induced a progressive increase in O_2_^−^ and H_2_O_2_ levels in radish cotyledons (Figure 6A–C). O_2_^−^ levels experienced a significant elevation to 6.65–8.12 nmol·g^−1^ FW following treatment with 0.25–2 mM Cs. H_2_O_2_ content displayed a clear upward trend in response to Cs treatment, reaching a maximum of 0.96 μmol·g^−1^ FW under 2 mM stress conditions, representing an increase of 8.45–47.70% compared to the control (Figure 6E,F). When the Cs treatment concentration reached 1.5 mM, the MDA content significantly increased compared to the control (*p* < 0.05), and reached its peak at 2 mM Cs treatment (8.42 μmol·g^−1^ FW) (Figure 6D). Superoxide dismutase (SOD) activity exhibited a concentration-dependent response, increasing by 133.03–136.64% at Cs levels ≥1 mM compared to the control (Figure 6A). Peroxidase (POD) activity showed threshold activation, with a 78.61–140.35% enhancement observed at Cs concentrations >1.5 mM (Figure 6B). Catalase (CAT) activity demonstrated a biphasic response, peaking at 1132.62 U·g^−1^ FW under 0.5 mM Cs treatment before declining gradually, though remaining at 61.66–93.35% above control levels across higher concentrations (Figure 6C).

### 2.6. Transcriptome Analysis of Radish Seedlings

**Figure 7:**
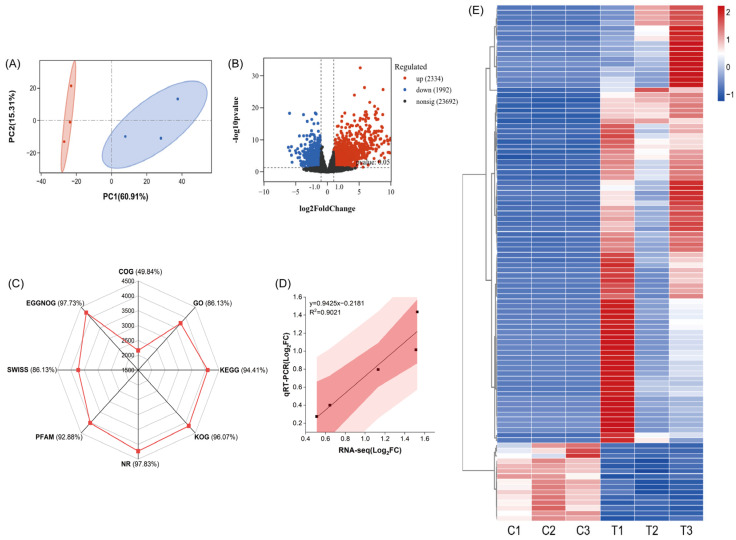
Annotation of transcriptome data and analysis of gene expression patterns in radish seedlings’ cotyledons under 2 mM Cs treatment. (**A**) Principal component analysis (PCA) plot. (**B**) Volcano map of DEGs. (**C**) Annotation degree of DEGs. (**D**) Correlation Analysis of qRT-PCR and RNA-seq; red area indicates 95% confidence intervals. (**E**) Heat map Analysis. C1–C3 indicate the control group, T1–T3 indicate the 2 mM Cs treatment group (*n* = 3). Red represents up-regulated genes; blue represents down-regulated genes (*n* = 3).

**Figure 8:**
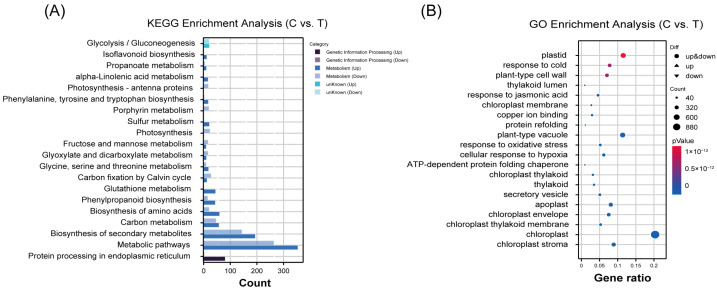
KEGG and GO enrichment analysis of radish seedlings’ cotyledons after 48 h of 2 mM Cs treatment. (**A**) Gene Ontology (GO) enrichment analysis of DEGs. The abscissa represents the proportion of the genes in the corresponding entry in all the genes in that entry, and the ordinate represents the different gene function entries. Circle size represents enrichment in the corresponding bar. Color represents enrichment significance, and circles indicate that the gene function is associated with both up-regulated genes and down-regulated genes. (**B**) Kyoto Encyclopedia of Genes and Genomes (KEGG) enrichment analysis of DEGs. The abscissa represents the pathway names, and the ordinate is the number of enriched genes (*n* = 3).

**Figure 9:**
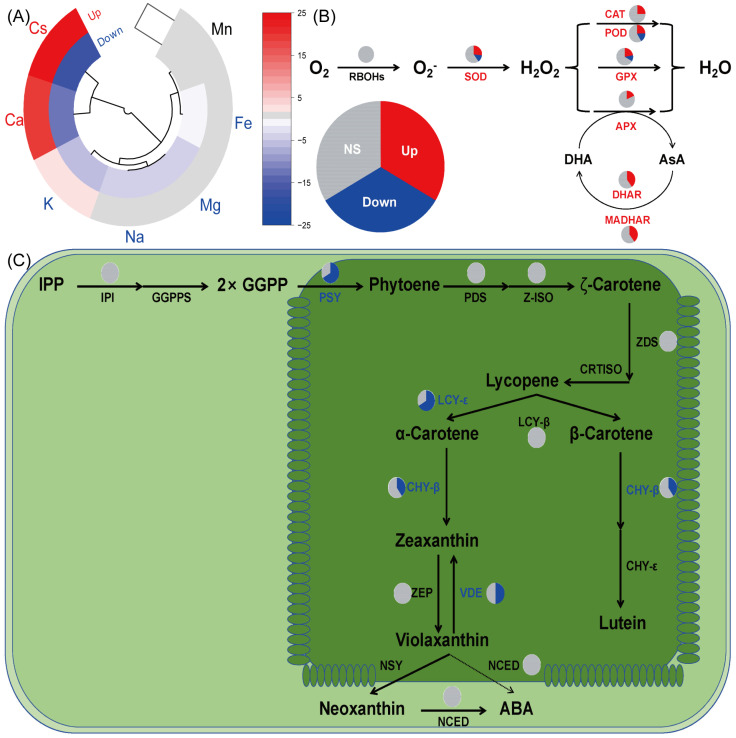
Visual analysis of genes involved in mineral elements absorption (**A**), antioxidant system (**B**), and carotenoid biosynthesis (**C**) in radish seedlings after 48 h of Cs exposure. Legend number indicates the number of genes. GPX: glutathione peroxidase, APX: ascorbate peroxidase, DHAR: dehydroascorbate reductase, MADHAR: monodehydroascorbate reductase (MDHAR), DHA: docosahexaenoic acid, AsA: L-ascorbic acid, IPP: isopentenyl pyrophosphate, IPI: isopentenyl diphosphate isomerase, GGPPS: two-geranyl pyrophosphate synthetase, GGPP: two-geranylgeranyl diphosphate, PSY: phytoene synthase, PDS: phytoene desaturase, Z-ISO: ζ-carotene isomerase, ZDS: ζ-carotene desaturase, CRTISO: carotenoid isomerase, LCY-α: lycopene α-cyclase, LCY-β: lycopene β-cyclase, CHY-β: β-ring hydroxylase, CHY-ε: ε-ring hydroxylase, ZEP: zeaxanthin epoxidase, VDE: violaxanthin de-epoxidase, NSY: neoxanthin synthase, NCED: 9-cis-epoxycarotenoid dioxygenase. In the pie chart, red represents up-regulated genes, blue represents down-regulated genes, and gray represents no significant difference for this gene. The figures were mapped based on the following KEGG metabolic pathways/GO term: GO: 0016209 (antioxidant activity), ko00906 (Carotenoid biosynthesis) (*n* = 3).

Transcriptome profiling of radish cotyledons under 2 mM Cs stress revealed comprehensive transcriptional reprogramming (Figure 7, Figure 8 and Figure 9). Principal component analysis (PCA) demonstrated clear separation between control and treated samples along the first principal component (PC1) (Figure 7A). Comparative analysis identified 4326 differentially expressed genes (DEGs), comprising 2334 up-regulated (e.g., *CSD1*, *FSD1*, *FSD2*) and 1992 down-regulated (e.g., *ABCG32*, *ABCG33*, *ABCF5*) transcripts, while 23,692 genes maintained stable expression profiles (Figure 7B). Functional annotation revealed 97.83% of DEGs could be mapped to the NCBI non-redundant (NR) protein database, representing the highest annotation rate among all databases analyzed. The heat map of cluster analysis of gene expression revealed distinct transcriptional signatures across treatment groups (Figure 7E), the detailed data can be found in Table S3. The key enzyme synthesis genes in the reactive oxygen metabolism pathway (*MSD1*, *CAT1*, *CAT2*, *POD34*, and *POD47*) were selected for qRT-PCR verification, as these genes showed significant differential expression in the transcriptome data. The validation experiments showed that gene expression demonstrated strong concordance with RNA-seq data (R^2^ = 0.9021, *p* < 0.05) (Figure 7D), confirming the reliability of transcriptome data for downstream analysis.

Gene Ontology (GO) enrichment analysis of DEGs in Cs-stressed radish cotyledons revealed significant enrichment across three functional categories: biological process, cellular component, and molecular function (Figure 8A). The top ten enriched terms were dominated by chloroplast-related components, including chloroplast stroma, thylakoid membrane, envelope, and thylakoid, along with other critical cellular structures and processes (apoplast, secretory vesicle, ATP-dependent protein folding chaperone, and cellular response to hypoxia). This indicated that Cs stress primarily targets chloroplast integrity and function, subsequently affecting photosynthesis and protein homeostasis (Table S4).

Kyoto Encyclopedia of Genes and Genomes (KEGG) pathway analysis further demonstrated that Cs stress significantly impacts multiple metabolic pathways (Figure 8B). The top ten enriched pathways included primary metabolic processes (carbon metabolism, Calvin cycle), secondary metabolite biosynthesis, and stress-responsive pathways (glutathione metabolism, sulfur metabolism). Notably, photosynthesis-related pathways (antenna proteins, photosynthetic pathway) were prominently affected, along with protein processing in the endoplasmic reticulum. These results collectively demonstrate that Cs stress disrupts photosynthetic efficiency, protein synthesis, and secondary metabolism, while activating cotyledon antioxidant and chelation systems (Table S5).

The DEGs in response to Cs stress in radish seedling cotyledons were further visualized to verify the above experimental results (Table S6). Transcriptional regulation of ion transporters under Cs stress revealed coordinated downregulation of Cs and Ca transporter genes and reduced expression of K, Na, Mg, Fe, and Mn transporter genes (Figure 9A), consistent with ion content measurements (Figure 5). The enzymatic antioxidant system showed comprehensive up-regulation, with increased expression of genes encoding SOD, CAT, POD, glutathione peroxidase (GPX), ascorbate peroxidase (APX), dehydroascorbate reductase (DHAR), and monodehydroascorbate reductase (MDHAR) (Figure 9B). This transcriptional activation corresponds with elevated antioxidant enzyme activities and subsequent membrane lipid peroxidation, as evidenced by MDA accumulation (Figure 6). In the carotenoid biosynthesis pathway, down-regulation of phytoene synthase (PSY) and lycopene ε-cyclase (LCY-ε) genes coupled with reduced expression of β-carotene hydroxylase (CHY-β) genes (Figure 9C) explained the observed decrease in cotyledon carotenoid content (Figure 4C). These molecular-level changes confirm and extend previous physiological observations, providing mechanistic insights into Cs-induced photoprotective pigment degradation.

### 2.7. Antioxidant Enzyme-Associated Gene Expression in Radish Seedlings to Cs Stress

**Figure 10:**
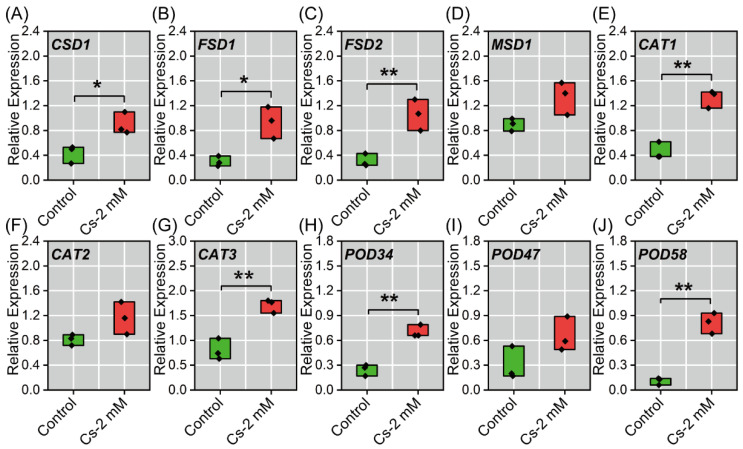
Effects of Cs stress on the antioxidant enzyme-associated gene expression in radish seedlings. (**A**) *CSD1* (copper/zinc superoxide dismutase 1) relative expression under Cs stress, (**B**) *FSD1* (iron superoxide dismutase 1) relative expression under Cs stress, (**C**) *FSD2* (iron superoxide dismutase 2) relative expression under Cs stress, (**D**) *MSD1* (manganese superoxide dismutase 1) relative expression under Cs stress, (**E**) *CAT1* (catalase 1) relative expression under Cs stress, (**F**) *CAT2* (catalase 2) relative expression under Cs stress, (**G**) *CAT3* (catalase 3) relative expression under Cs stress, (**H**) *POD34* (peroxidase 34) relative expression under Cs stress, (**I**) *POD47* (peroxidase 48) relative expression under Cs stress, (**J**) *POD58* (peroxidase 58) relative expression under Cs stress. * and ** represent significant differences in different treatment groups and the control at the 0.05 and 0.01 levels (*n* = 3).

Quantitative reverse transcription PCR (qRT-PCR) analysis revealed differential regulation of antioxidant-related genes in response to 2 mM Cs exposure. While transcript levels of *MSD1*, *CAT2*, and *POD47* remained statistically unchanged (*p* > 0.05, Figure 10D,F,I), which might be due to the differential expression of these genes in other organs or the presence of isoforms, significant up-regulation was observed for several key genes. The cytosolic superoxide dismutase gene *CSD1* and iron superoxide dismutase gene *FSD1* exhibited moderate induction, showing 1.84-fold (*p* < 0.05) and 3.12-fold (*p* < 0.05) increases relative to controls, respectively (Figure 10A,B). More pronounced transcriptional activation was detected for *FSD2* (240.86%, *p* < 0.01), *POD34* (185.14%, *p* < 0.01), *POD58* (118.89%, *p* < 0.01), and *CAT3* (112.45%, *p* < 0.01), all demonstrating highly significant upregulation (Figure 10C,E,G,J) compared to the control. These results suggested compartment-specific oxidative stress responses, with particular activation of iron-dependent superoxide dismutase isoforms and specific peroxidase genes, potentially indicating their crucial roles in Cs-induced ROS detoxification.

## 3. Discussion

In this study, it was found that Cs significantly inhibited radish seedlings’ growth, and reduced the biomass of roots, stems, and cotyledons, while suppressing root elongation (Figure 1). These findings align with previous reports in *Arabidopsis thaliana* [32] and other plants [10,13,17]. Notably, we observed decreased carotenoid content, but no cotyledon etiolation and chlorophyll decreased (Figure 1 and Figure 4A,B), which suggests radish may possess unique mechanisms to maintain photosynthetic pigment stability under Cs stress (Figure 4). Calcium ions (Ca^2+^), as important second messengers in plants, work together with calmodulin to affect chlorophyll synthesis [33,34]. In this study, the Cs stress did not cause the cotyledons of radish seedlings to etiolate (Figure 1), and the chlorophyll content remained stable (Figure 4A,B). This might be related to the up-regulation of Ca^2+^ and its transport proteins.

The accumulation pattern of Cs in radish exhibited preferential distribution aboveground (cotyledon > stem > root, Figure 2). This distribution pattern exhibited similarities to *Brassica juncea*, *Cucumissativus* L., and *Amaranth tricolor* [10,11,12,13]. Quantitative analysis revealed an inverse relationship between BCF and Cs exposure, while TF demonstrated a positive correlation with increasing Cs levels (Figure 2). Notably, while the BCF decreased with increasing Cs concentrations, the overall cesium accumulation capacity of the plants exhibited enhancement. This apparent contradiction implies that the radish still exhibited a high absorption efficiency when exposed to high concentrations of Cs (Figure 2), and the observed decline in BCF is primarily driven by root biomass reduction, rather than a reduction in Cs absorption (Figure 1). Furthermore, the TF remained stable across 1.5 mM to 2 mM Cs exposures (Figure 2), suggesting minimal interference of Cs with plant transport protein functionality [12]. The observed TF enhancement under elevated Cs enrichment aligns with previously reported patterns in *Cucumis sativus* L. and *Amaranthus tricolor* [10,13].

The decline in Pn of plants under heavy metal stress is generally attributed to two distinct mechanisms: stomatal limitations and non-stomatal limitations [35,36,37]. Results revealed that the decrease in Pn was primarily due to non-stomatal limitation, as evidenced by the inconsistent changes in Gs and Ci (Figure 3A–D). However, under 0.25–1 mM Cs treatment, the trends of Gs and Ci became similar, suggesting that some stomatal limitation factors still influenced Pn [37]. Tr remained consistent with Gs (Figure 3B,D), indicating that the underlying mechanism for Tr variation was mainly related to Gs [35,37]. Chlorophyll fluorescence parameters provided valuable insights into plant responses to external stressors. There were no significant changes observed in Fv/Fm, Fv’/Fm’, ΦPSII, and qP under Cs treatment (Figure 4D–I), which is similar to the results of previous studies [13,17,22,23], suggesting that the Photosystem II complex (PSII) structure was not significantly compromised by Cs exposure [23]. In chloroplasts, electron transport is tightly coupled with ATP synthesis [37]; the increase in ETR implied that Cs treatment could provide additional energy for the Calvin-Benson cycle. However, the lack of a significant change in Pn indicated that Rubisco enzyme activity was likely limited [38]. This suggests that while electron transport was enhanced, the downstream fixation of carbon was constrained. Excessive light energy can lead to the production of ROS [39,40], which can damage cellular structures. Consistent with this, the content of H_2_O_2_ increased under Cs treatment, and MDA, a marker of membrane structure damage, also rose significantly, indicating that cell membrane integrity was compromised (Figure 6D,F). The reduction in NPQ was closely related to changes in zeaxanthin content [13,41]. Transcriptomic data revealed down-regulation of gene expression associated with zeaxanthin synthesis (e.g., LCY-ε, CHY-β, VDE) (Figure 9C), and the content of carotenoid decreased (Figure 4C), which corroborated the observed decrease in this compound. This likely contributed to oxidative damage in radish cotyledons, as reduced NPQ implies diminished dissipation of excess light energy as heat [35,42]. Furthermore, no significant changes in qP were detected (Figure 4H). The activities of SOD, POD, and CAT all increased to a certain extent under Cs treatment (Figure 6B–D), and the expression of related antioxidant enzymes genes (e.g., *CSD1, FSD1, FED2*; Figure 10) also increased indicating that excess light energy was primarily converted into ROS and then scavenged by antioxidant enzymes [37]. Simultaneously, less light energy was dissipated as heat, suggesting one of the protective mechanisms of PS II in radish seedlings.

The disruption of mineral homeostasis represents a critical aspect of Cs phytotoxicity in plants. Our results reveal significant depletion of essential elements (K, Na, Mg, Fe) in radish tissues (Figure 5), consistent with previous findings in *Vicia faba* and *Brassica juncea* [12,25,43,44]. Studies have shown that Cs can affect the absorption of mineral elements, especially K, which may be due to its effect on the expression of genes related to mineral absorption and thus on the absorption of external elements by plants [12,25,43,44]. The competitive inhibition between Cs^+^ and K^+^ through shared transport systems (AKT, HAK, KUP) [14,45,46,47] explains the observed K^+^ depletion. The decreasing trend of Na content is similar to that of K content (Figure 5A,B), which is mainly caused by the decrease of the aboveground part. It was found that Na, K, and Cs share part of the ionic channel proteins, such as ABCG, KIR, VDCC, and RCA [16,45,48]. Therefore, it can be considered that the decrease of Na and K content is due to the competitive absorption effect caused by the addition of Cs, and the toxicity of Cs to radish should not be ignored. Meanwhile, Na^+^ and K^+^ also have a competitive absorption relationship within the plant (with cotransporters such as AKT1, KUP/HAK, etc.). This might be one of the reasons for the decrease in the content of Na^+^ and K^+^. Fe is involved in a variety of physiological and biochemical reactions, such as plant photosynthesis and the oxidation–reduction process, and is an essential element of the plant body [49,50,51]. Generally, Fe deficiency in plants will lead to cotyledon etiolation and a decrease in chlorophyll content [52,53]. However, this phenomenon was not found in radish under Cs stress, which may be due to the fact that Fe content mainly decreased in the underground part of the radish rather than the aboveground part. The expression of Fe-related superoxide dismutase genes *FSD1* and *FSD2* was significantly increased (Figure 10), so it is speculated that Cs could poison the proteins mediating iron absorption in radish roots [49,50], which affected the absorption of Fe in radish but did not affect the transport of Fe in the plant, thus leading to a significant decrease in Fe content in the underground part (Figure 5). In addition, Fe uptake is also likely impaired by Fe chelation disruption. Interestingly, despite upregulation of Ca^2+^ transporters in cotyledons, whole-plant Ca^2+^ content remained stable, indicating tissue-specific redistribution rather than net accumulation.

In radish, Cs induced oxidative stress triggers a complex antioxidant response. ROS homeostasis is crucial in plants [26,54], and this study focused on the enzymatic antioxidant system to investigate how Cs induces ROS production, activates antioxidant enzymes, and causes cell membrane damage. Our results revealed a concentration-dependent activation sequence: H_2_O_2_ and O_2_^−^ were induced upon 0.25 mM Cs treatment, which in turn activated antioxidant enzymes. At 1 mM Cs treatment, O_2_^−^ reached its peak level, allowing SOD to achieve its highest activity as the enzyme specialized in scavenging O_2_^−^, thereby converting a large amount of O_2_^−^ into H_2_O_2_ and leading to a gradual increase in H_2_O_2_ content [55] (Figure 6). It was thus speculated that low concentrations of Cs would cause a burst of ROS, inducing antioxidant enzymes (primarily CAT) to scavenge the excess ROS. However, at high Cs concentrations, CAT activity is inhibited due to its toxicity, necessitating increased POD and SOD activities to counteract ROS [26]. At 1.5 mM Cs treatment, ROS levels were elevated, but the antioxidant system had reached its scavenging limit, unable to remove additional ROS independently, resulting in cell damage and a gradual increase in MDA content (Figure 6). This graded response suggests that CAT is the primary ROS scavenger at lower Cs concentrations, while POD and SOD provide secondary protection. This pattern was confirmed by relative antioxidant enzyme gene expression and transcriptome analysis, which showed up-regulation of key antioxidant genes such as *POD58*, *CAT1*, *APX1*, *GPX3*, and others (Figure 6 and Figure 10), consistent with previous reports [26,56]. The final accumulation of MDA indicates the saturation of the antioxidant system and provides a quantitative threshold for Cs toxicity in radish. The findings highlight that Cs treatment triggers ROS accumulation, particularly H_2_O_2_, leading to oxidative damage and potential disruption of cell membrane integrity. Such oxidative stress responses may contribute to broader physiological impacts on plant health under prolonged exposure to Cs-induced stress.

Comprehensive transcriptome analysis (Figure 7, Figure 8 and Figure 9) reveals that Cs impacts multiple physiological processes in radish seedlings, particularly photosynthesis and protein metabolism. It has been found that Cs can disrupt the abscisic acid signaling pathway, ROS metabolism, and HSP family proteins in *Arabidopsis thaliana* [57,58], thereby disrupting its growth. However, there are still few reports on the physiological response of radish to Cs. The transcriptome results showed that Cs could affect photosynthesis, protein synthesis, and the antioxidant system in radish (Figure 7), which was consistent with the experimental results, proving that the transcriptome analysis was reliable. In a previous study, Cs could affect the photosynthesis of *Amaranth tricolor* [13], but its molecular mechanism was not elucidated, and it was found that Cs could cause abnormal expression of genes (e.g., *psaB*, *psbC*, *LHCA1*) related to the photosynthesis pathway, blocking the electron transport process from plastoquinone-QA to plastoquinone-QB, resulting in inhibiting photosynthesis in *Brassica juncea* [23]. Our work provides mechanistic insights through chloroplast-related gene expression patterns and carotenoid biosynthesis pathway analysis (Figure 1 and Figure 10C). The up-regulation of *FSD1* genes (Figure 10), known to participate in photosynthetic electron transport [59,60], suggests a compensatory response to Cs-induced photosynthetic inhibition. These findings expand our understanding of Cs phytotoxicity mechanisms and provide potential biomarkers for assessing Cs stress in plants.

## 4. Materials and Methods

### 4.1. Plant Culture and Cs Treatment

Radish (*Raphanus sativus* ‘Nanpanzhou’) seeds were purchased from Minyuan Seed Co., Ltd. (Dangyang, China), and Cesium chloride (CsCl, analytical reagent) was used as the source of Cs. Radish seeds were disinfected with 70% ethanol and soaked in ultrapure water for 24 h. Then, they were transferred to a Petri dish with filter paper and placed in the culture chamber for germination. When the seeds germinated to a root length of about 1 cm, they were transferred to the colonization basket [15,61]. The seedlings were cultured in tissue culture flasks (200 mL/bottle) for 7 days and then transferred to modified Hoagland nutrient solution containing different concentrations of Cs [0.25, 0.5, 1.0, 1.5, and 2 mM] for 48 h; untreated plants grown in Hoagland solution served as controls. Photographic documentation of seedling morphology was conducted after 48 h of Cs exposure, with six biological replicates per treatment group. The specific culture conditions and Hoagland solution composition are shown in Table S1.

### 4.2. Determination of Physiological Parameters

The Cs-treated radish seedlings were soaked in 20 mM EDTA-Na_2_ for 20 min and washed three times with distilled water. After being dried with filter paper, the plants were divided into roots, stems, and cotyledons, and then weighed. Cotyledon rings were made from plant cotyledons using a 1 cm^2^ hole punch, mixed 1:1 (*v*/*v*) with 95% ethanol and 80% acetone, and shaken for 24 h in the dark to extract chloroplast pigments. The light absorption values at 663 nm, 645 nm, and 470 nm were measured, and the chlorophyll a (Chl a), chlorophyll b (Chl b), and carotenoid (Car.) contents were calculated [62].

#### 4.2.1. Determination of Element Content and Photosynthetic Parameters

The weighed samples were dried in an air-drying oven (80 °C), and the dry weight was measured. Then, the sample was placed into a digestive tube, and 10 mL of digestion solution [HNO_3_+HClO_4_ (3:1, *v*/*v*)] was added; the sample was digested and then filtered with a 0.45 μm water system filter membrane (Jinteng experimental equipment Co., Ltd., Tianjin, China). The filtered samples were determined for elemental content by flame atomic absorption spectrophotometry (TAS-990, Beijing Purkinje GENERAL Instrument Co., Ltd., Beijing, China) [56,61,63].

The net photosynthetic rate (Pn), stomatal conductance (Gs), intercellular CO_2_ concentration (Ci), and transpiration rate (Tr) in the fourth true cotyledon were measured by the portable photosynthesis system (GFS-3000, WALZ, GER) [37]. Measurements were taken between 8:30 and 11:30, and six biological replicates were performed for each group (*n* = 6). The plants were moved to a dark room at night, where the dark-adapted maximum fluorescence (Fm), dark-adapted minimum fluorescence (Fo), and steady-state fluorescence (Fs) were measured the next morning by a pulse-modulated fluorometer (FMS-2, Hansatech, UK) during actinic illumination with 1500 mmol·m^−1^s^−1^ photons. After the plants were transferred back to the constant temperature illumination culture chamber and exposed to light for 1 h, the maximum fluorescence (Fm’) and minimal fluorescence (Fo’) in the light-adapted state were measured. Then, data of the maximum photochemical efficiency (Fv/Fm), capture efficiency of excitation energy (Fv’/Fm’), electron transfer rate (ETR), photochemical quenching coefficient (qP), non-photochemical quenching coefficient (NPQ), and actual photochemical efficiency (ΦPSII) in photosystem II (PSII) were calculated according to previous reports [64].

#### 4.2.2. Determination of the ROS Metabolism

First, 0.2 g samples were placed in a mortar with quartz sand, and 5 mL of PBS (50 mM, pH 7.8) was added. After grinding into the homogenate on ice, the mixture was centrifuged at 7000 rcf and 4 °C for 20 min. The activities of superoxide dismutase (SOD), peroxidase (POD), and catalase (CAT) in the supernatant were determined by the tetrazolium blue (NBT) photoreduction method, ultraviolet colorimetry, and guaiacol method, respectively. Superoxide dismutase (SOD) activity was determined spectrophotometrically at 560 nm, with one unit of enzyme activity (U) defined as the amount of enzyme required to inhibit the photochemical reduction of 50% NBT, representing one unit of fresh weight (U·g^−1^ FW). Catalase (CAT) activity was determined spectrophotometrically at 240 nm, with one unit of enzyme activity (U) defined as the amount of enzyme required to increase the absorbance by 0.01 per minute per gram of fresh weight (U·g^−1^ FW). Peroxidase (POD) activity was determined spectrophotometrically at 470 nm, with one unit of enzyme activity (U) defined as the amount of enzyme required to increase the absorbance by 0.1 per minute per gram of fresh weight (U·g^−1^ FW) [26,44,65].

Malondialdehyde (MDA) content was measured by grinding the samples in 10% Trichloroacetic acid (TCA) and centrifuging at 4000 rpm for 10 min. Then, 2 mL of the supernatant was reacted with 2 mL of 0.6% thiobarbituric acid (TBA) in a boiling water bath (98 °C) for 15 min. The reaction products of MDA and TBA showed maximum light absorption at 532 nm, while the reaction products of soluble sugar and TBA also showed maximum light absorption at 532 nm (maximum light absorption wavelength was 450 nm). Therefore, OD_450_, OD_532_, and OD_600_ were measured to exclude the interference of soluble sugar, and the concentration of MDA in the reaction system was calculated using MDA content = 6.45 × (OD_532_ − OD_600_) − 0.56 × OD_450_ [66]

Next, weigh 1 g of radish cotyledons and homogenize in 2 mL of PBS (50 mM, pH 7.8) under ice-bath conditions. Transfer the homogenate to a centrifuge tube and centrifuge at 10,000 rpm for 20 min at 4 °C. Collect 0.5 mL of the supernatant, then add 0.5 mL of PBS and 1.5 mL of 1 mM hydroxylamine hydrochloride. Vortex for 30 s, then add 2 mL of 17 mM pamino-benzenesulfonic acid and 2 mL of 7 mM α-naphthylamine. Vortex for another 30 s, then incubate at 25 °C for 20 min. Measure the absorbance at 530 nm. For the standard curve, use NaNO_2_ as a substitute for superoxide anion (O_2_^−^) to prepare solutions with concentrations of 5, 10, 15, 20, 25, and 30 nmol·mL^−1^ [67].

The H_2_O_2_ content determination procedure involved weighing approximately 1 g of plant material and grinding it with 5 mL of 0.1% TCA on ice to form a homogenate, which was then centrifuged at 12,000 rcf for 15 min. One milliliter of the supernatant was transferred, mixed with 1 mL each of 0.1 M PBS and 1 M KI, vortexed, and incubated at 28 °C for 1 h before measuring the absorbance at 390 nm (OD_390_). For standard curve preparation, varying volumes of H_2_O_2_ stock solution are mixed with 0.1% TCA to achieve concentrations ranging from 0 to 0.07 mM, with each sample containing 1 mL of PBS and KI [68].

Diaminobenzidine (DAB) and nitrotetrazolium blue chloride (NBT) are, respectively, specific histochemical dyes for H_2_O_2_ and O_2_^−^; the staining results reflected the distribution of H_2_O_2_ and O_2_^−^ in plant tissues, providing supplementary functions for the determination of their contents in physiological experiments [69,70,71]. The radish cotyledons were cut out and put into 0.1% DAB and 0.1% NBT staining solution, respectively. After 30 min of shaking, the radish cotyledons were placed into a vacuum pump for vacuum staining for 3 h. After 3 h, the radish cotyledons were placed into a shaker for 20 min, and the staining solution was recovered. After the decolorization was completed, they were placed into the scanner for scanning, avoiding light throughout the staining process, and six replicates were taken for each treatment concentration [72,73].

### 4.3. Transcriptome and Gene Expression Analysis

After 48 h of Cs treatment, samples were washed three times with ddH_2_O. Cotyledons were immediately placed in liquid nitrogen, and total RNA was extracted using TRIzol reagent (Invitrogen, USA) according to the manufacturer’s instructions. Using transcriptome sequencing methods, differentially expressed genes (DEGs) were screened at a *p*-value < 0.05 and log2|FC| ≥ 1. The screened DEGs were analyzed in the Kyoto Encyclopedia of Genes and Genomes (KEGG) and Gene Ontology (GO) databases. Transcriptome sequencing was performed by Beijing Tsingke Biotechnology Co., Ltd. (Beijing, China) and was limited to the company; three biological replicates were performed in each group [74,75,76].

Radish cotyledons were cut, and total RNA was extracted using TRNzol Universal Total RNA Extraction reagent (Tiangen Biochemical Technology Co., Ltd., Beijing, China), as detailed in the instructions (https://www.tiangen.com/). The concentration and quality of RNA were evaluated by agarose gel electrophoresis and a micro-nucleic acid detection system, and then reverse transcribed into cDNA using the TSINGKE TSK322S SynScript™ III cDNA Synthesis Mix kit (Tsingke Biotechnology Co., Ltd., Beijing, China). Finally, for quantitative reverse transcription polymerase chain reaction (qRT-PCR) amplification, using specific primers, the PCR reagents were from Beijing branch Biological Technology Co., Ltd.’s 2 × T5 FastqPCR Mix (SYBR Green I); for the detailed steps, see the instructions (https://www.tsingke.com.cn/) [77]. Gene expression was calculated using the method 2^−∆∆Cq^ [78,79] and *H3* and *ACT7* were the primers for qRT-PCR; the primer sequences for each gene are shown in Table S2.

### 4.4. Statistical Analysis

The basic experimental data were collected by Microsoft Excel 2021 (Microsoft Inc., Redmond, WA, USA), and SPSS 26.0 was used to test the normality hypothesis of the experimental data (Mean ± Standard Error). In the study, the physiological experiments were replicated 6 times (*n* = 6), and the transcriptional replicate samples were 3 (*n* = 3). The Shapiro–Wilk test assessed data normality (*p* > 0.05), and a subsequent significance test was performed. For groups that were 2 (N = 2), a *t*-test was performed. For groups (N) that were greater than 2 (N > 2), one-way analysis of variance (ANOVA) and least significant difference (LSD) tests were performed [80,81]. Using the biological cloud platform (https://cloud.tsingke.com.cn/) allowed the visualization analysis of differentially expressed genes. Graphics were drawn using Origin 2024 software (Origin Lab. Corporation, Northampton, MA, USA) and merged using Adobe Illustrator CC 2019 software (Adobe Systems Incorporated, San Jose, CA, USA).

## 5. Conclusions

The present study comprehensively investigated the physiological and molecular responses of radish (*Raphanus sativus* L.) to Cs stress, revealing three key aspects of Cs impact and plant adaptation mechanisms. Firstly, radish exhibits distinct Cs accumulation patterns, with preferential translocation to cotyledons rather than roots, and concentration-dependent accumulation correlating with external Cs levels. Secondly, Cs exposure triggered multiple physiological disruptions in radish seedlings: (1) significant growth inhibition, particularly in root development and biomass accumulation; (2) altered photosynthetic pigments metabolism, specifically reducing carotenoid content, photosynthesis was not significantly affected; membrane lipid peroxidation evidenced by MDA accumulation, particularly at higher Cs concentrations (>1.5 mM); (3) disruption of mineral homeostasis, characterized by increased Ca translocation to shoots and decreased accumulation of K, Na, Mg, and Fe. At last, at the molecular level, Cs stress induced activation of the enzymatic antioxidant system, showing sequential induction of CAT, POD, and SOD activities in a concentration-dependent manner, up-regulation of antioxidant-related genes (*FSD1*, *FSD2*, *POD58*, *CAT1*) and stress-responsive pathways.

The present study demonstrated that radish exhibited stable biomass, normal physiological metabolism, and strong Cs absorption capacity under ≤1 mM Cs exposure, as a potential candidate for phytoremediation. Future research should evaluate Cs removal efficiency across the entire growth cycle in in situ contaminated environments to validate their applicability. Additionally, integrating genetic engineering and biochemical techniques can further elucidate the roles of differentially expressed genes identified herein in enhancing Cs tolerance and accumulation, deepening the comprehension of plant radioactive nuclide enrichment mechanisms.

## Data Availability

Data available upon request.

## Supplementary Materials

The following supporting information can be downloaded at: https://www.mdpi.com/article/10.3390/plants14121802/s1, Table S1: Culture conditions; Table S2: Primer sequences for qRT-PCR; Table S3: Supplementary information for heatmap. Table S4: Supplementary information for GO enrichment analysis; Table S5: Supplementary information for KEGG enrichment analysis; Table S6: Expression of each gene in the gene expression pattern.
