# Supplementary material for: Cesium Accumulation Patterns and Stress Response in Hydroponic Radish (Raphanus sativus L.): A Physiological–Transcriptomic Study
Source: Plants (Basel). 2025 Jun 12;14(12):1802. doi: 10.3390/plants14121802 (PMC12196763; doi:10.3390/plants14121802)
Supplement: Supplementary file 1 [file plants-14-01802-s001.zip › plants-3682301-supplementary.pdf]

Cesium accumulation patterns and stress response in hydroponic radish (*Raphanus sativus* L.): A physiological-transcriptomic study

Yu-han Wen [a †], Xi Chen [b †], Ming Sun [a], Chao-hui Yang [a], Meng-yuan Xu [a], Feng-xiang Lai [a], Si-qi Fu [a], Yu-meng Fan [a], Xin-peng Guo [a], Qun Li[a], Guo Wu [a *]

[a] College of Life Science, Sichuan Normal University, Chengdu, 610101, China.

[b] College of Resources and Environmental Sciences, Nanjing Agricultural University, Nanjing, 210095, China.

[†] Both authors contributed equally to this work.

[*] Corresponding author.
Guo Wu
Email: **wuguoyk@sicnu.edu.cn** (G.Wu)
Tel./Fax: +86-28-84480656
College of Life Science, Sichuan Normal University, Chengdu 610101, Sichuan, China.

Supplementary Results

Table S1. Culture conditions

Indicators	Conditions
Hongland Nutrient Solution	1 mM KNO_3
	1 mM $Ca(NO_3)_2 \cdot 4H_2O$
	0.4 mM $MgSO_4 \cdot 7H_2O$
	0.2 mM NH_4NO_3
	4 μm EDTA-Fe
	4.5 μM $MnCl_2 \cdot 4H_2O$
	0.38 μM $ZnSO_4$
	0.1 μM $CuSO_4 \cdot 5H_2O$
	0.25 μM $H_2MoO_4 \cdot 4H_2O$
	pH 5.5 – 5.8
Temperature	25-30 ℃
Light intensity	3500 lx, 16 h light /8 h dark cycle
Relative Humidity	70-80%

Table S2. Primer sequences for qRT-PCR

Gene	Forward Primers (5' to 3')	Reverse Primers (5' to 3')
*H3**	CAAGACTGACCTGCGTTTCC	CTCTCCTCTGATCCTGCGAG
*ACT7**	GATGGGTCAGAAAGATGC	CTGTTGGCTTTAGGGTTA
CSD1	ATGGCTGCTCCTCACACCTTC	CGGAGACGACAGTCAGCGATT
FSD1	GTTACAACAATGGCGACCTCCT	TGAGTGGCAGCAGCAGCAT
FSD2	CGCCGTAACGAAGAAGAGAACA	CGCAGTTGGAACACCTGATGG
MSD1	CCATCACCAGAAGCACCACCAG	ATGACCTCCGCCGTTGAACTTG
CAT1	AGGACTACAGGCACATGGATGG	GGCAGCGATGGAGTCATAGAGA
CAT2	CAACTCTGGTGCTCCTGTATGG	CCTTCGCACTGGCTCCTCTA
CAT3	TGCTCACCACAACAATCACCAT	ACCTATCTCCAGCCTGCTTGAA
POD34	GCATCCTTCGTCTTCACTTCC	TCCTATTCATCGCCTCCACAA
POD47	CCTGCGAATCTGAGTCTAC	GAATCTGGTGTGGTAAGTCT
POD58	AGATGGTCGCACGGCTAA	TCGTCGCAATGTTCGTAAGA

"*" indicts internal reference gene.

Table S3. Supplementary information for heatmap.

GeneID	C1(C)	C2(C)	C3(C)	T1(T)	T2(T)	T3(T)
gene-LOC108808280	4.419647	5.03358	2.165416	78.394669	34.24007	48.886627
gene-LOC108811614	51.629719	67.637001	62.574867	9.723864	12.367716	17.327059
gene-LOC108840190	0.188005	0.103315	0.14527	12.438921	3.425195	39.268192
gene-LOC108854635	0.129867	0.21367	0.412278	5.141841	4.942574	6.562023
gene-LOC108845113	1.610497	2.169125	2.736587	45.677681	15.690324	63.019493
gene-LOC108841017	0	0.181214	0	6.331817	20.777822	49.674675
gene-LOC108828688	0.030848	0	0.037155	125.548553	2.671913	20.81369
gene-LOC108818705	4.563209	4.48555	8.934344	863.11615	114.770988	318.327698
gene-LOC108821536	3.484669	2.654436	1.038599	107.68222	50.839817	660.289062
gene-LOC108816757	123.620468	119.9739	107.736053	777.598572	422.290558	836.973267
gene-LOC108840278	390.645355	327.288574	712.07959	104.741402	71.681976	111.254173
gene-LOC108847431	12.463842	13.169216	13.039464	47.782963	47.523193	40.573029
gene-LOC108844727	3.29868	6.278929	2.535851	769.868652	100.786484	237.39238
gene-LOC108847973	5.682115	5.057631	5.19103	82.65699	532.656006	699.845825
gene-LOC108847980	5.586766	4.732863	4.878111	77.483116	464.255371	607.784241
gene-LOC108846989	0.156408	0.128264	0.470967	27.081579	6.229107	28.391003
gene-LOC108844578	0.670999	0.848135	0.253889	22.373518	7.049094	41.549191
gene-LOC108848730	6.520503	5.72238	3.506018	60.149853	35.517784	132.663513
MSTRG.12136.2	0.363546	0.332768	0.283071	4.798305	3.195819	3.371557
gene-LOC108852127	5.922895	7.887097	12.667006	0.055599	0.263932	0.120754
gene-LOC108852447	1.400417	1.168391	0.236705	53.684525	12.356722	100.811584

gene-LOC108851904	3.129081	1.611099	2.707315	58.470711	17.025154	37.145073
gene-LOC108848435	9.074502	9.013177	3.416759	569.237366	55.232735	420.66748
gene-LOC108852780	0.167693	0.188692	0	59.404125	4.226582	37.249626
gene-LOC108849716	0.177062	0.164469	0.342121	50.982895	6.677382	18.920345
gene-LOC108855102	21.158695	27.308886	27.51679	7.758538	8.368156	7.91041
gene-LOC108849203	11.821465	11.847874	11.058532	4.123913	2.166255	3.487377
gene-LOC108861648	0	0	0	26.385979	2.873102	4.042989
gene-LOC108857458	7.589753	11.558332	13.095131	66.546249	50.7356	66.151634
gene-LOC108857526	11.656493	12.233102	12.293664	176.325256	63.193066	139.206345
gene-LOC108859815	0.269141	0.233139	0.288389	43.678658	3.684484	18.057056
gene-LOC108857469	0.310709	0.164423	0.385142	161.88591	4.695019	20.265842
gene-LOC108861622	6.630526	6.986963	6.427518	2.1999	2.184722	2.671915
gene-LOC108856253	89.003075	101.925095	90.246635	49.733379	36.5163	39.454121
gene-LOC108862574	0.441016	0.439669	0.601845	70.056953	5.062708	52.459076
gene-LOC108862719	0.478309	0.512909	0.142283	59.729969	5.924197	17.843773
gene-LOC108856202	0.499326	0.169611	0.342434	172.04631	5.218211	21.486229
gene-LOC108856567	1.552315	1.451593	1.669607	10.260088	5.566992	10.24718
gene-LOC108861295	2.042584	1.140523	3.175128	44.16925	43.962673	58.623291
gene-LOC108861294	1.875535	1.034968	2.535188	999.649719	55.126377	300.455536

Continued to **Table S3**

**GeneID**	**C1(C)**	**C2(C)**	**C3(C)**	**T1(T)**	**T2(T)**	**T3(T)**
gene-LOC108812705	8.329611	7.051417	9.125463	109.945023	36.645672	110.71759

gene-LOC108813009	0	0	0	9.411824	4.741314	1.172942
gene-LOC108810166	0.09564	0.237149	0.230841	125.257324	9.497021	44.519283
gene-LOC108812096	0.485643	0.452339	0.384242	110.850853	12.396563	44.247135
gene-LOC108813560	0.108959	0.057918	0	27.974199	5.726494	9.374127
gene-LOC108809026	55.031235	69.799545	62.679859	29.998758	23.403358	26.902418
gene-LOC108813872	4.491363	5.547738	1.595292	69.085388	110.032761	377.10849
gene-LOC108813699	52.243896	69.785309	37.68037	491.160522	1476.228638	1781.353638
gene-LOC108811793	3.750422	5.629985	4.327922	0.358385	0.750166	0.565991
gene-LOC108811837	0.827017	0.63748	0.832073	86.306526	19.135599	58.835735
gene-LOC108812385	0.09747	0.088263	0.079496	22.153753	2.427288	6.088296
gene-LOC108806779	25.592459	24.749195	25.152651	97.355202	66.045998	105.928268
gene-LOC108808900	17.870394	26.501446	17.801994	3.850744	2.538356	1.795889
gene-LOC108810841	1.764159	1.0438	0.877268	9.853116	10.966226	12.006019
gene-LOC108811487	14.129787	11.73063	11.715669	47.246227	44.000816	58.372787
gene-LOC108813785	0.104194	0.436826	0.220541	63.261166	4.918022	12.271565
gene-LOC108814828	16.80043	19.181238	17.557966	9.68164	8.651146	10.312756
gene-LOC108817148	0.118645	0.222362	0.097991	11.182954	3.748824	13.957027
gene-LOC108818451	15.21725	17.504559	16.079308	87.24707	63.279446	141.946625
gene-LOC108816114	0.059028	0.310195	0.18591	10.525035	3.785451	16.003683
gene-LOC108816103	12.034629	16.387112	13.151932	3.120698	3.310414	2.256241
gene-LOC108817054	5.292824	4.183784	4.048681	41.918762	24.350786	44.386192
gene-LOC108817415	0.044883	0.051264	0.066096	14.835258	0.837899	5.075351

gene-LOC108817200	0.252142	0.250581	0.585074	20.706242	12.721193	91.103142
gene-LOC108815789	0.360957	0.411946	0.418248	1.723571	2.165574	2.434945
gene-LOC108817231	0.163386	0.641897	0.673868	9.785237	82.04483	602.051697
gene-LOC108817316	15.554268	22.642439	17.809162	140.340256	90.470482	100.230003
gene-LOC108814733	0.096843	0.11045	0.050786	8.248493	2.144959	2.537071
gene-LOC108819691	0.725535	0.675366	0.76981	36.546429	7.073726	53.514271
gene-LOC108820793	384.856171	505.487427	467.72406	163.994598	94.925888	115.219688
gene-LOC108826894	7.041574	3.799987	12.291825	160.633224	59.46629	176.704086
gene-LOC108824432	0.343067	0.076567	0.070079	12.704339	25.994402	127.36422
gene-LOC108823993	1.539951	0.319327	0.808531	22.923233	205.611221	486.334595
gene-LOC108827782	4.614367	11.250728	6.729328	397.679718	129.861893	1293.863403
gene-LOC108827781	18.222242	18.67944	10.617995	151.796021	416.747589	786.161865
gene-LOC108827526	0.17577	0.150853	0.138736	24.869318	4.529844	12.617777
gene-LOC108827424	0.068273	0.11193	0.049461	13.214321	21.987381	102.425774
gene-LOC108826163	22.94997	23.063692	17.861389	3.848795	5.237435	6.589241
gene-LOC108823377	9.880984	18.982283	21.190319	2.12575	1.209277	1.042756
gene-LOC108823288	0.056396	0	0.035666	31.374231	1.226131	18.430735
gene-LOC108827671	0.339984	0.356072	0.465929	67.369354	5.332591	9.889528

Continued to **Table S3**

**GeneID**	**C1(C)**	**C2(C)**	**C3(C)**	**T1(T)**	**T2(T)**	**T3(T)**
gene-LOC108825486	1.802063	0.864755	2.874014	66.912659	17.244286	75.961052
gene-LOC108828933	10.269125	10.163151	11.846846	170.978928	54.395866	220.866074

gene-LOC108829639	0.205744	0.241834	0.496799	21.186647	5.425593	5.132221
gene-LOC108830019	0.17908	0.260304	0.087536	12.569054	5.829196	4.827537
gene-LOC108830068	0.133458	0.426661	0.116249	95.352547	12.787618	40.344158
gene-LOC108830077	0.216712	0.174923	0.22809	12.713015	5.569418	9.48961
gene-LOC108830415	0.163717	0.098956	0.487043	212.929749	3.600227	23.870613
gene-LOC108831767	0.062758	0.128052	0	53.413086	3.039499	48.56604
gene-LOC108833063	10.091393	1.599654	5.183971	103.539055	817.753418	1120.125244
gene-LOC108834541	0.144815	0.138917	0.447247	5.111499	11.152045	7.659501
gene-LOC108836039	0.390556	0.26083	0.122906	13.350128	60.104198	348.17514
gene-LOC108836085	2.278116	2.591028	1.937732	131.847595	24.734957	34.059738
gene-LOC108836689	0.447161	0.515278	0.332455	224.025772	10.990771	63.814964
gene-LOC108837952	0.291394	0.248905	0	99.970459	5.929791	41.812897
gene-LOC108838407	1.806778	1.773398	1.815595	25.978199	13.362907	48.944275
gene-LOC108838791	0.265775	0.183331	0.037786	58.651112	2.985067	16.503555
gene-LOC108839128	0.077412	0	0	10.653406	6.643806	9.790444
gene-LOC108816090	0	0.035565	0.133431	41.779556	3.128557	27.847654

Table S4. Supplementary information for GO enrichment analysis

ID	Discription	Gene ratio	Count	p-value
GO:0009570	chloroplast stroma	0.0890 (385/4326)	385	7.54591E-52
GO:0009507	chloroplast	0.2032 (879/4326)	879	1.7524E-51
GO:0009535	chloroplast thylakoid membrane	0.0529 (229/4326)	229	1.23952E-38
GO:0009941	chloroplast envelope	0.0754 (326/4326)	326	3.62884E-34
GO:0048046	apoplast	0.0811 (351/4326)	351	3.74511E-32
GO:0099503	secretory vesicle	0.0509 (220/4326)	220	2.10183E-26
GO:0009579	thylakoid	0.0349 (151/4326)	151	1.16018E-25
GO:0009534	chloroplast thylakoid	0.0317 (137/4326)	137	1.75922E-25
GO:0140662	ATP-dependent protein folding chaperone	0.0099 (43/4326)	43	3.26559E-16
GO:0071456	cellular response to hypoxia	0.0617 (267/4326)	267	3.54239E-16
GO:0006979	response to oxidative stress	0.0520 (225/4326)	225	1.18413E-14
GO:0000325	plant-type vacuole	0.1137 (492/4326)	492	1.83595E-14
GO:0042026	protein refolding	0.0113 (49/4326)	49	6.13596E-14
GO:0005507	copper ion binding	0.0294 (127/4326)	127	9.29498E-14
GO:0031969	chloroplast membrane	0.0273 (118/4326)	118	1.34826E-13
GO:0009753	response to jasmonic acid	0.0460 (199/4326)	199	1.45233E-13
GO:0031977	thylakoid lumen	0.0095 (41/4326)	41	8.73789E-13
GO:0009505	plant-type cell wall	0.0705 (305/4326)	305	8.88165E-13
GO:0009409	response to cold	0.0779 (337/4326)	337	9.01915E-13
GO:0009536	plastid	0.1153 (499/4326)	499	1.42564E-12

Table S5. Supplementary information for KEGG enrichment analysis

ID	Discription	Gene ratio	Count	p-value
ko01100	Metabolic pathways	0.1429 (618/4326)	618	1.55118E-12
ko01110	Biosynthesis of secondary metabolites	0.0781 (338/4326)	338	1.25667E-11
ko01200	Carbon metabolism	0.0243 (105/4326)	105	8.42262E-09
ko04141	Protein processing in endoplasmic reticulum	0.0194 (84/4326)	84	3.584E-08
ko00196	Photosynthesis - antenna proteins	0.0042 (18/4326)	18	1.92252E-07
ko00710	Carbon fixation by Calvin cycle	0.0095 (41/4326)	41	2.31147E-07
ko00480	Glutathione metabolism	0.0109 (47/4326)	47	5.20626E-07
ko00920	Sulfur metabolism	0.0055 (24/4326)	24	9.09759E-06
ko00943	Isoflavonoid biosynthesis	0.0030 (13/4326)	13	1.98376E-05
ko00195	Photosynthesis	0.0060 (26/4326)	26	3.77618E-05
ko00260	Glycine, serine and threonine metabolism	0.0067 (29/4326)	29	0.000438458
ko00010	Glycolysis / Gluconeogenesis	0.0099 (43/4326)	43	0.000959924
ko00051	Fructose and mannose metabolism	0.0062 (27/4326)	27	0.001665579
ko00860	Porphyrin metabolism	0.0049 (21/4326)	21	0.00203409
ko01230	Biosynthesis of amino acids	0.0187 (81/4326)	81	0.002459794
ko00940	Phenylpropanoid biosynthesis	0.0139 (60/4326)	60	0.002566085
ko00592	alpha-Linolenic acid metabolism	0.0039 (17/4326)	17	0.002626122
ko00630	Glyoxylate and dicarboxylate metabolism	0.0065 (28/4326)	28	0.003656354
ko00640	Propanoate metabolism	0.0037 (16/4326)	16	0.005644369
ko00400	Phenylalanine, tyrosine and tryptophan biosynthesis	0.0049 (21/4326)	21	0.006208894

Table S6. Expression of each gene in the gene expression pattern

Function	Gene Discription	Regulation		
		Up	Down	Non-sign
Elements absorption	Cs absorption	25	17	197
	K absorption	2	6	36
	Ca absorption	19	12	129
	Na absorption	0	5	27
	Mg absorption	0	5	40
	Fe absorption	0	1	11
	Mn absorption	0	0	4
Antioxidant system	RBOHs	0	0	4
	SOD	4	2	9
	POD	13	9	31
	CAT	2	0	6
	GPX	2	1	6
	APX	2	0	9
	DHAR	2	0	3
	MADHAR	2	0	3
Carotenoid biosynthesis	IPI	0	0	3
	GGPPS	-	-	-
	PSY	0	2	1
	PDS	0	0	4
	Z-ISO	0	0	1
	CRISTO	-	-	-
	ZDS	0	0	1
	LCY-ε	0	2	1
	LCY-β	0	0	1
	CHY-β	0	2	3
	CHY-ε	-	-	-
	ZEP	0	0	1
	VDE	0	1	1
	NSY	-	-	-
	NCED	0	0	3

"-" indicts the related genes were not screened.
